# Polymorphisms in metabolic genes, their combination and interaction with tobacco smoke and alcohol consumption and risk of gastric cancer: a case-control study in an Italian population

**DOI:** 10.1186/1471-2407-7-206

**Published:** 2007-11-08

**Authors:** Stefania Boccia, Fakhredin A Sayed-Tabatabaei, Roberto Persiani, Francesco Gianfagna, Stefano Rausei, Dario Arzani, Antonio La Greca, Domenico D'Ugo, Giuseppe La Torre, Cornelia M van Duijn, Gualtiero Ricciardi

**Affiliations:** 1 Genetic Epidemiology and Molecular Biology Unit, Institute of Hygiene, Università Cattolica del Sacro Cuore, Rome, Italy; 2 Medicines Evaluation Board Agency, The Hague, The Netherlands; 3 Department of Surgery, Università Cattolica del Sacro Cuore, Rome, Italy; 4 Department of Epidemiology & Biostatistics, Erasmus Medical Center, Rotterdam, The Netherlands

## Abstract

**Background:**

The distribution and the potential gene-gene and gene-environment interaction of selected metabolic genetic polymorphisms was investigated in relation to gastric cancer risk in an Italian population.

**Methods:**

One hundred and seven cases and 254 hospital controls, matched by age and gender, were genotyped for *CYP1A1*, *CYP2E1*, *mEH*, *GSTM1*, *GSTT1*, *NAT2 *and *SULT1A1 *polymorphisms. Haplotype analysis was performed for *EPHX1 *exons 3 and 4, as well as *CYP2E1 Rsa*I (*5 alleles) and *CYP2E1 Dra*I (*5A or *6 alleles). The effect modification by alcohol and cigarette smoking was tested with the heterogeneity test, while the attributable proportion (AP) was used to measure the biological interaction from the gene-gene interaction analysis.

**Results:**

Gastric cancer risk was found to be associated with the inheritance of *GSTT1 *null genotype (OR = 2.10, 95%CI: 1.27–3.44) and the *SULT1A1 *His/His genotype (OR = 2.46, 95%CI: 1.03–5.90). No differences were observed for the haplotype distributions among cases and controls. For the first time an increased risk was detected among individuals carrying the *6 variant allele of *CYP2E1 *if ever-drinkers (OR = 3.70; 95%CI: 1.45–9.37) with respect to never-drinkers (OR = 0.18; 95% CI: 0.22–1.46) (*p *value of heterogeneity among the two estimates = 0.001). Similarly, the effect of *SULT1A1 *variant genotype resulted restricted to ever-smokers, with an OR of 2.58 (95%CI: 1.27–5.25) for the carriers of His allele among smokers, and an OR of 0.86 (95%CI: 0.45–1.64) among never-smokers (*p *value of heterogeneity among the two estimates = 0.03). The gene-gene interaction analyses demonstrated that individuals with combined *GSTT1 *null and *NAT2 *slow acetylators had an additional increased risk of gastric cancer, with an OR of 3.00 (95%CI: 1.52–5.93) and an AP of 52%.

**Conclusion:**

*GSTT1*, *SULT1A1 *and *NAT2 *polymorphisms appear to modulate individual's susceptibility to gastric cancer in this Italian population, particularly when more than one unfavourable genotype is present, or when combined with cigarette smoke. The increased risk for the carriers of CYP2E1*5A or *6 alleles among drinkers need to be confirmed by larger prospective studies.

## Background

Gastric cancer is the second most common cause of mortality from cancer, with 647,000 deaths reported worldwide in 2002 [1]. In many populations, particularly in high-income countries, in the last decades its incidence has gradually decreased, however it still represents the fifth most common type of cancer in Europe and the fourth internationally [1]. The development of gastric cancer appears to be the result of a complex interaction between lifestyle and genetic factors. Among the lifestyle and related risk factors, *Helicobacter pylori *infection, tobacco smoking, a high intake of salt and lack of food refrigeration all seem to play a major role [2]. Additionally, gastric cancer shows a familial clustering [3]. With regards to genetic factors, several Single Nucleotide Polymorphisms (SNPs) might potentially alter the individual susceptibility to gastric cancer, among them genes coding for metabolic enzymes [4].

A major part of carcinogenic substances require metabolic activation by enzymes to be genotoxic, and inherited variations in carcinogens metabolizing genes may alter enzyme activity and subsequently carcinogens activation or deactivation. Phase I enzymes, including Cytochrome P450 (CYP) and microsomal Epoxide Hydrolase (mEH), activate several compounds to form genotoxic electrophilic intermediates. Activated metabolites are then, in part, detoxified by phase II enzymes, such as glutathione S-transferase (GST), N-acetyltransferase (NAT) and Sulfotransferase (SULT) [5]. We recently showed, for the first time, that *SULT1A1 *Arg213His polymorphism might affect the risk of gastric cancer [6], while contradictory results concerning several SNPs in metabolic genes have been reported [7-15].

Based on the knowledge that metabolic genes are presumed to modulate an individual's susceptibility to cancer by interacting with carcinogens, and since the inheritance of several unfavourable genotypes is supposed to additionally increase the risk of gastric cancer [8,9,11], this hospital-based case-control study aims to investigate the effect on gastric cancer of selected SNPs of *CYP1A1*, *CYP2E1*, *mEH*, *GSTM1*, *GSTT1*, *NAT2*, *SULT1A1*, and their differential effect according to tobacco smoking and alcohol habits. We also investigated to what extent the inheritance of more than one unfavourable genotype affects the risk of gastric cancer.

## Methods

### Study population

The study subjects were selected according to a case-control study design as previously described [16]. Briefly, cases were consecutive primary gastric adenocarcinoma patients, with histological confirmation, who underwent a curative gastrectomy in the "A. Gemelli" teaching hospital, located within the Università Cattolica del Sacro Cuore in Rome. We defined gastric cancer cases as including International Classification of Disease Ninth revision codes 151.0–151.9. Controls were selected from cancer-free patients, with a broad range of diagnoses including around 15% of blood donors, admitted to the same hospital during the identical time period and were frequency matched to cases for age (± 5 years) and gender. All subjects were Caucasians born in Italy. According to the Lauren histotype classification [17], the majority (57.8%) of the gastric cancer cases were intestinal. The tumours were located in the antrum (39.3%), in the corpus (14.8%), in the antrum/corpus (28.0%), in the cardia (10.3%), stumps (5.6%) and in the fundum (2.0%). Based on the cytological and architectural atypisms, as well as the histo-pathological reports [18], patients' tumours were classified accordingly: 68.3% scarcely differentiated, 29.2% moderately differentiated, 2.5% well-differentiated, while 53.8% were staged I–II and 46.2% staged III–IV. With a response rate of 95% and 90% respectively for cases and controls, 102 gastric cancer and 254 controls were recruited.

A venous blood sample was drawn from each participant, collected into an EDTA-coated tubes from which DNA was isolated from peripheral blood lymphocytes. The study was approved by the local review board and written informed consent was obtained from each subject. The procedures followed were in accordance with the Helsinki Declaration.

### Data collection

Cases and controls were interviewed by trained medical doctors using a standard questionnaire to elicit information on demographic variables, tobacco smoking (including cigarette, cigar and pipe) and drinking history, dietary habits and family history of cancer. Questions pertaining to lifestyle focused on the time period ending one year prior to diagnosis. Smoking status was categorized as never and ever-smokers (including both current and former smokers) and alcohol consumption as drinkers/non-drinkers (the latter including individuals whose alcohol intake less than 7 g/day). Fruit and vegetables intake was classified as high if the participant consumed at least two portions of fruit and two portions of vegetables per day. Meals salt addition was referred to the use of adding salt to the entrées during the main meals. Family history (including non-melanoma skin cancer) of cancer referred to parents, siblings and offspring. Data concerning previous *Helicobacter pylori *infection was not available for either cases or controls. The response rate for completing the interview was 99.1% for cases (106/107) and 99.6% (253/254) for controls, with the exception of data relating to a family history of cancer [unknown in 7.4% (8/107) of cases and 3.5% (9/254) of controls].

### Genotyping

*GSTM1 *and *GSTT1 *null alleles were identified using a multiplex-Polymerase Chain Reaction (PCR)-based method as described by Arand et al. [19]. The polymorphic site at nucleotide 638 in exon 7 (Arg213His) of the *SULT1A1 *gene was genotyped by PCR-Restriction Fragment Length Polymorphisms (RFLP) analysis as described by Coughtrie et al. [20]. Identification of the *mEH *exon 3 (Tyr113His) and exon 4 (His139Arg) polymorphisms was performed using a RFLP-based method [21]. *CYP1A1 *3'-flanking region *Msp*I polymorphism (CYP1A1*2A allele), *CYP2E1 Rsa*I polymorphism (CYP2E1*5 alleles) and *CYP2E1 Dra*I (*5A or *6 alleles) were also determined by PCR-RFLP analyses (21). Three known slow acetylator alleles, NAT2*5A, *6A and *7A were identified as previously described by Peluso et al [22]. Fast acetylator genotypes are the homo-heterozyogous wild-type alleles (*4A), slow acetylator genotypes are those with 2 slow acetylator alleles [23]. Quality control for each genotyping was performed in each experiment, and 10% of the total samples were randomly selected and reanalyzed with 100% concordance. The analyst was blinded to the case or control status of the samples.

### Statistical analysis

The relationship between gastric cancer and putative risk factors were measured using the adjusted odds ratios (ORs) and their 95% CI derived from logistic regression analysis using STATA software (version 8.2). We considered possible risk factors for gastric cancer as potential confounders if the addition of that variable to the model changed the OR by 10% or greater. Confounding checks were performed in both of the univariate and final multivariate models. If a factor was identified as a confounder of any estimated main effect, it was kept in all models. Based on these criteria, we controlled for age, gender, alcohol consumption and family history of cancer, when appropriate. In the multivariable model, we adjusted for the continuous variables of age and alcohol (g/day).

The genotypes of *GSTM1 *and *GSTT1 *were dichotomized according to the presence *versus *absence of the null allele, and *NAT2 *was dichotomized according to the inferred phenotype (slow *versus *fast). We analyzed exon 3 and exon 4 *mEH *genotypes by "imputed phenotype" as suggested from Smith and Harrison [24]. Lastly, we conducted haplotype analysis for *EPHX1 *exons 3 and 4, as well as CYP2E1*5 and *5A or *6 using Cocaphase software. Hardy-Weinberg Equilibrium (HWE) was tested for separately all of the case and control SNPs.

In order to assess if the effect of the studied polymorphisms is modified by tobacco smoking and alcohol consumption, we performed a stratified logistic regression analysis. An heterogeneity test was then used to test differences among the strata.

Biological interaction between two genes was estimated using departure from additivity of effects as the criterion of interaction, as suggested by Rothman [25]. To quantify the amount of interaction, the attributable proportion (AP) due to interaction was calculated as described by Andersson et al [26]. The AP due to interaction is the proportion of individuals among those exposed to the two interacting factors that is attributable to the interaction per se and it is equal to 0 in the absence of a biological interaction [25]. Finally, in order to test for more than multiplicative effect among two genes, the likelihood ratio test was used, with the homozygous wild-type individuals for both genes as the reference group.

## Results

General characteristics of the study population are presented in Table 1. Alcohol consumption and family history of cancer were associated with an increased risk of gastric cancer, with ORs of 2.10 (95% CI: 1.22–3.60) and an OR of 1.93 (95%CI: 1.14–3.26), respectively (Table 1). The genotype frequencies of our control group were in line with those for Caucasians and were in HWE both for cases and controls (*p *> 0.05) [5,20]. As shown in Table 2, we found a significant difference in the distribution of *GSTT1 *null and *SULT1A1 *His/His genotype amongst cases and controls: 37.1% *versus *22.4% (OR = 2.10, 95% CI: 1.27–3.44) and 10.3% *versus *5.1% (OR = 2.46; 95% CI: 1.03–5.90), respectively. An increased risk was also detected for *NAT2 *slow acetylators (OR = 1.38, 95% CI: 0.88–2.19), however not statistically significant. Haplotype analyses indicated that there was no significant linkage disequilibrium between *EPHX1 *exons 3 and 4, as well as CYP2E1*5 and *5A or *6, amongst the cases and the controls. Furthermore, the frequency of the estimated haplotypes was the same among the groups (data not shown).

**Table 1:** Odds Ratios (95% CI) of gastric cancer according to the collected variables and their frequency distribution among 107 cases and 254 controls

	Cases % (n)	Controls % (n)	OR (95% CI) †
Age (years ± SD)	66.4 ± 12.0	64.0 ± 12.8	-
Male gender	52.3 (56)	55.5 (141)	-
Alcohol drinkers			
Non-drinkers°	29.9 (32)	47.6 (121)	1*
Drinkers	70.1 (73)	52.4 (133)	2.10 (1.22–3.60)
Smoking status			
Never	53.3 (57)	57.5 (146)	1*
Ever	46.7 (50)	42.5 (108)	1.10 (0.64–1.90)
Fruit and vegetables intake			
High‡	20.0 (21)	15.8 (40)	1*
Low	80.2 (85)	84.2 (213)	0.95 (0.50–1.90)
Grilled meat			
≤ 2 times/month	52.3 (56)	48.4 (123)	1*
> 2 times/month	47.7 (61)	51.6 (131)	0.99 (0.60–1.66)
Meals salt addition^			
No	85.1 (91)	92.9 (235)	1*
Yes	14.9 (16)	7.1 (18)	1.70 (0.78–3.67)
Familiarity for cancer			
No	61.6 (61)	78.4 (192)	1*
Yes	38.4 (38)	21.6 (53)	1.93 (1.14–3.26)
Familiarity for gastric cancer			
No	88.0 (88)	94.4 (237)	1*
Yes	12.0 (12)	5.6 (14)	1.88 (0.80–4.44)

* Reference category; † OR adjusted for age, gender, alcohol consumption and familiarity for cancer; ‡ High fruit and vegetables consumption is defined as at least 2 portions of fruit and 2 portions of vegetables per day; ° Non-drinkers defined as an alcohol intake less than 7 g/day; ^ adding salt to the entrées during the main meals

**Table 2:** Odds Ratios (95% CI) of gastric cancer for SNPs in metabolic genes and their frequency distribution among 107 cases and 254 controls

		Cases % (n)	Controls % (n)	OR (95% CI) * †
*GSTM1 null*		56.2 (59)	52.7 (135)	1.13 (0.71–1.79)
*GSTT1 null*		37.1 (39)	22.4 (57)	2.10 (1.27–3.44)
CYP1A1*2A		20.5 (22)	22.0 (56)	0.88 (0.50–1.54)
CYP2E1*5		4.7 (5)	7.8 (20)	0.54 (0.20–1.50)
CYP2E1*5A or *6		14.5 (15)	10.6 (27)	1.33 (0.67–2.65)
*NAT2 *Slow ‡		59.8 (64)	51.8 (131)	1. 38 (0.88–2.19)
*SULT1A1*	Arg/His	36.5 (39)	33.5 (85)	1.35 (0.82–2.21)
	His/His	10.3 (11)	5.1 (13)	2.46 (1.03–5.90)
*EPHX1 *exon 3	Tyr/His	38.7 (41)	36.0 (90)	1.24 (0.76–2.04)
	His/His	14.1 (15)	11.2 (28)	1.37 (0.67–2.80)
*EPHX4 *exon 4	His/Arg	30.5 (32)	37.4 (95)	0.77 (0.47–1.27)
	Arg/Arg	5.7 (6)	2.4 (6)	2.28 (0.70–7.20)
Imputed *mEH *phenotypes ^	Rapid	15.8 (15)	23.7 (59)	0.60 (0.30–1.15)
	Slow	25.3 (24)	21.2 (54)	1.00 (0.55–1.78)
	Very slow	8.4 (8)	8.6 (21)	0.82 (0.33–2.00)

* OR adjusted for age and gender

† Reference groups are the homozygous wild genotypes for each gene

‡ Reference group is fast acetylators (homo-heterozygous for the wild-type allele)

^ Reference group is the normal imputed phenotype

From the stratified analysis according to smoking status (Table 3), the significant association for *SULT1A1 *observed in the overall analysis seems to be limited to ever smokers, with a *p *value for heterogeneity among the two strata of 0.03 (Table 3). On the other hand, the increased risk for *GSTT1 *null individuals was significant regardless of the smoking status (Table 3). As for the effect modification by alcohol habits, drinking subjects carrying the variant allele of CYP2E1 (*5A or *6 alleles) had an OR of 3.70 (95%CI: 1.45–9.37) of gastric cancer compared to those drinking without the variant allele, with the result of the heterogeneity test among the strata showing a significant effect modification by alcohol (*p *value = 0.001, Table 3).

**Table 3:** Odds Ratios (95% CI) of gastric cancer for SNPs in metabolic genes according to smoking status and alcohol habits

	Never-smokers (57 cases, 146 controls)		Ever-smokers (50 cases, 108 controls)		*p *for heterogeneity
	cases/controls	OR (95% CI) * †	cases/controls	OR (95% CI)	
	
*GSTM1 *null	33/70	1.55 (0.83–2.90)	26/64	0.70 (0.35–1.39)	0.10
*GSTT1 *null	21/34	2.09 (1.06–4.11)	18/23	2.17 (1.02–4.59)	0.92
CYP1A1*2A	14/33	1.09 (0.53–2.27)	8/23	0.66 (0.27–1.61)	0.40
CYP2E1*5	4/11	0.86 (0.26–2.88)	1/9	0.20 (0.02–2.70)	0.24
CYP2E1*5A or *6	10/17	1.59 (0.67–3.79)	5/10	0.99 (0.31–3.13)	0.58
*NAT2 *Slow ‡	34/77	1.39 (0.74–2.60)	30/54	1.50 (0.75–2.98)	0.87
*SULT1A1 *His carriers	22/62	0.86 (0.45–1.64)	27/36	2.58 (1.27–5.25)	0.03
*EPHX1 *exon 3 His carriers	28/62	1.31 (0.70–2.46)	28/56	1.12 (0.57–2.22)	0.69
*EPHX4 *exon 4 Arg carriers	21/63	0.84 (0.44–1.60)	16/38	0.89 (0.43–1.85)	0.85

	Never-drinkers (32 cases, 121 controls)		Ever-drinkers (73 cases, 133 controls)		*p *for heterogeneity

	cases/controls	OR (95% CI)	cases/controls	OR (95% CI)	
	
*GSTM1 *null	16/64	0.95 (0.43–2.10)	43/70	1.23 (0.69–2.20)	0.55
*GSTT1 *null	13/24	3.15 (1.32–7.47)	26/33	1.72 (0.92–3.22)	0.27
CYP2E1*5	0/10	-	5/10	0.86 (0.28–2.68)	-
CYP2E1*5A or *6	1/19	0.18 (0.22–1.46)	14/8	3.70 (1.45–9.37)	0.001
*SULT1A1 *His carriers	16/51	1.42 (0.63–3.17)	33/47	1.56 (0.86–2.82)	0.98

* OR adjusted for age and gender

† Reference groups are the homozygous wild genotypes for each gene

‡ Reference group is fast acetylators (homo-heterozygous for the wild-type allele)

To reduce the chance of multiple testing, we limited the gene-gene interaction analyses to the three SNPs that exhibited the most prominent association with gastric cancer. It was observed that in all of the combinations individuals carrying two risk genotypes had an additional risk compared to those with only one risk genotype, with an AP greater than 0, however there was no evidence of multiplicative interaction (*p *values > 0.05, Table 4). The observed effect was particularly high amongst individuals with both *GSTT1 *null and *NAT2 *slow (OR = 3.00, 95% CI: 1.52–5.93; AP = 52%) (Table 4). Additionally, by stratifying these data according to smoking status (data not shown), ever-smoker individuals with combined *GSTT1 *null and *NAT2 *slow had an OR of 4.23 (95% CI: 1.49–12.01) compared to ever-smokers with combined normal variants, while an OR of 2.60 (95% CI: 1.00–6.67) appeared using the same comparators amongst never-smokers (*p *value of heterogeneity among the two estimates = 0.49).

**Table 4:** Age and gender adjusted Odds Ratios (95% CI) of gastric cancer for selected gene-gene interaction analyses

		*GSTT1*		*SULT1A1*	
		Present	Null	Arg/Arg	His car.
*NAT2*	Fast	1*	1. 38 (0.63–3.01)	1*	1.45 (0. 71–2.95)
	cases/controls	30/93	13/29	23/74	20/48
	Slow	1.07 (0.61–1.88)	3.00 (1.52–5.93)	1.40 (0. 75–2.60)	2.00 (1.03–3.89)
	cases/controls	36/103	26/28	35/81	29/50
	
		*p *for interaction† = 0.17		*p *for interaction = 0.97	
		AP‡ = 52%		AP = 8%	

*SULT1A1*	Arg/Arg	1*	1.53 (0.86–2.71)	-	-
	cases/controls	35/122	22/34		
	His carriers	2.30 (1.18–4.45)	2.87 (1.36–6.05)	-	-
	cases/controls	31/75	17/23		
		*p *for interaction = 0.70			
		AP = 1%			

* Reference category; † By likelihood ratio test; ‡ Attributable Proportion due to biological interaction (see methods)

## Discussion

This case-control study of 107 surgical cases of gastric adenocarcinoma and 254 controls born in Italy evaluated the effect on gastric cancer risk of several metabolic gene polymorphisms simultaneously. Results showed a significantly increased risk for *GSTT1 *null and for *SULT1A1 *homozygotes, and an additional risk for *NAT2 *slow acetylator individuals, although not statistically significant. Risks associated with those genes became substantive when two unfavourable genotypes were combined, with evidence of biological interaction between them. From the gene-environment interaction analysis, we showed effect modification of the association between *SULT1A1 *and gastric cancer by tobacco smoking, and CYP2E1 (*5A or *6 alleles) by alcohol drinking. In addition, our results confirm previous findings of gastric cancer risk to be increased by alcohol intake and family history for cancer [27,28].

Several limitations should be taken into account in the interpretation of our results. Firstly, based on the prevalence of the analyzed genotypic variants in our population (Table 2), our study was powered to detect an OR of 2.0 for common polymorphisms (with a significance level of 5%), however not for CYP2E1*5 allele carriers, CYP2E1*5A or *6 allele carriers and the homozygotes variants of *SULT1A1*, *EPHX3 *and *EPHX4*. The study's sample size limits the ability to explore the combined effects of the genotypes, or gene-environment interactions, which highlights the need to increase the sample size in order to confirm our results. However, when appropriately conducted, large and small studies should give, theoretically, the same results, with just a more precise effect measure estimate from the larger ones [29]. Secondly, as in all case-control studies information bias may exist, leading to biased ORs related to the gene-environment interaction results. Thirdly, data on *Helicobacter pylori *infection were not available in our population.

This is the first study conducted on an homogenous ethnic group who evaluated the effect on gastric cancer risk of several metabolic genes SNPs contemporarily, and the effect of their combination with tobacco and alcohol. One of the main source of confounding in the genetic association studies arises from population stratification, since the ethnicity itself may be related to a specific disease and to the allele frequencies as well [30,31]. Our study showed a significant association between *GSTT1 *null genotype and gastric cancer, which is in keeping with the results of a recent meta-analysis considering only high-quality papers [8]. Individuals who have the homozyogous deletion in *GSTT1 *have no enzyme activity, and thus are more susceptible to carcinogens such as benzo [α]pyrene-7,8-diol epoxide and smaller reactive hydrocarbons, such as ethylene oxide and diepoxybutane [8]. We also reported that individuals carriers of the *SULT1A1 *variant allele, who have limited detoxification capability of xeniobiotics through sulfonate conjugation, have an additional risk of gastric cancer if smokers.

To our knowledge, we reported for the first time a strong effect modification by alcohol of the association between CYP2E1*5A or *6 alleles and gastric cancer, with an increased risk among ever-drinkers. Two previous studies [32,33] reported no association between CYP2E1*5A or *6 allele and gastric cancer, however no one of them stratified data according to alcohol habits. Additionally, one study evaluating the identical association among black South-African males showed an increased risk of oesophageal cancer among drinkers carrying the CYP2E1*5A or *6 alleles [34]. CYP2E1 is a naturally ethanol-inducible enzyme that is mainly involved in the metabolic activation of *N*-nitrosamines present in tobacco smoke and some dietary compounds, for which a causative role in gastric carcinogenesis has been hypothesised [2], and in the metabolism of fatty acids and several halogenated and aromatic compounds [35]. Additionally, CYP2E1 plays a minor role in alcohol metabolism, through the oxidation of ethanol to acetaldehyde and 1-hydroxyacetyl radicals [35]. The *5A or *6 alleles of *CYP2E1 *is characterized by some studies in an increased gene expression [36], so that individuals carrying the unfavourable variant might be at higher risk of gastric cancer because of: i) hyper activation of *N*-nitrosamines in more reactive species, especially among drinkers since enzyme activity is induced by alcohol; ii) hyper production of reactive oxygen species and subsequent cell toxicity generated by ethanol metabolism among drinkers. We expected to gain similar results for *CYP2E1 Rsa*I polymorphism, identically associated with increased enzyme activity, however the few subjects in the stratified analysis probably did not show it. Since these results, however, are based on very few subjects (only one case drinker bearing *5A or *6 alleles) they need to be confirmed by larger studies.

Among the main results of our study, we found that *GSTT1 *null genotype individuals contemporarily *NAT2 *slow acetylators have a strongly increased risk of gastric cancer, with a more than just the additive effect of the risks associated with each of the two inherited SNPs. N-acetylation is considered a major detoxification step for carcinogenic aromatic arylamines, while *GSTT1 *is involved in the detoxification of polycyclic aromatic hydrocarbons, so individuals with one or both depleted phase II enzyme activities might be particularly susceptible to gastric damage from carcinogens, which is supported by the finding of an additional risk for ever-smokers. We used the attributable proportion due to interaction as a measure to quantify the biological interaction between those combined SNPs and showed a strong interaction between them. Assuming that the relationships studied are causal and based on the definition of biological interaction among two component causes [25,37], our results suggest that 52% of gastric cancer cases among *GSTT1 *null individuals with combined *NAT2 *slow acetylator phenotype are caused through a mechanism in which both risk factors are biological dependent in the same disease process. In other words, since biological interaction among two causes occurs when the effect of one is dependent from the presence of the other, in the absence of either of the two components (*GSTT1 *null or *NAT2 *slow), than a substantial number of gastric cancer cases would not occur. Given that in our population 25% of cases had a combination of those unfavourable genotypes, this means that a non negligible proportion of gastric cancer cases would have never developed if those enzymatic activities were adequate.

## Conclusion

This study suggests that in this Italian population, *GSTT1*, *SULT1A1 *and *NAT2 *polymorphisms may modulate an individual's susceptibility to gastric cancer, particularly when more than one unfavourable genotype is present and in combination with cigarette smoke. Additionally, we showed that individuals carrying the *5A or *6 alleles of *CYP2E1 *are at increased risk for gastric cancer in drinkers. Clearly, since our study is based on a limited number of cases, it is critical that larger prospective studies possibly based on a single ethnic group confirms our results.

## List of abbreviations used

AP = attributable proportion; CI = Confidence Interval; CYP = Cytochrome P450; GST = glutathione S-transferase; HWE = Hardy-Weinberg Equilibrium; mEH = microsomal Epoxide Hydrolase; NAT = N-acetyltransferase; OR = Odds Ratio; PCR = Polymerase Chain Reaction; RR = Risk Ratio or Rate Ratio; SULT = Sulfotransferase.

## Competing interests

The author(s) declare they have no competing interests.

## Authors' contributions

All authors read and approved the final manuscript. SB and GR conceived the study and coordinated the research group; SB and FG performed the statistical analysis and drafted the manuscript; FAST and GLT participated in the data analysis; DA carried out the genotyping; CMVD helped to draft the manuscript; RP, SR, DDU and ALG enrolled the patients and interviewed all them.

## Pre-publication history

The pre-publication history for this paper can be accessed here:


